# Identification of Host-Dependent Survival Factors for Intracellular *Mycobacterium tuberculosis* through an siRNA Screen

**DOI:** 10.1371/journal.ppat.1000839

**Published:** 2010-04-15

**Authors:** Shilpi Jayaswal, Md. Azhar Kamal, Raina Dua, Shashank Gupta, Tanmay Majumdar, Gobardhan Das, Dhiraj Kumar, Kanury V. S. Rao

**Affiliations:** Immunology Group, International Centre for Genetic Engineering and Biotechnology, New Delhi, India; Harvard School of Public Health, United States of America

## Abstract

The stable infection of host macrophages by *Mycobacterium tuberculosis* (Mtb) involves, and depends on, the attenuation of the diverse microbicidal responses mounted by the host cell. This is primarily achieved through targeted perturbations of the host cellular signaling machinery. Therefore, in view of the dependency of the pathogen on host molecules for its intracellular survival, we wanted to test whether targeting such factors could provide an alternate route for the therapeutic management of tuberculosis. To first identify components of the host signaling machinery that regulate intracellular survival of Mtb, we performed an siRNA screen against all known kinases and phosphatases in murine macrophages infected with the virulent strain, H37Rv. Several validated targets could be identified by this method where silencing led either to a significant decrease, or enhancement in the intracellular mycobacterial load. To further resolve the functional relevance of these targets, we also screened against these identified targets in cells infected with different strains of multiple drug-resistant mycobacteria which differed in terms of their intracellular growth properties. The results obtained subsequently allowed us to filter the core set of host regulatory molecules that functioned independently of the phenotypic variations exhibited by the pathogen. Then, using a combination of both *in vitro* and *in vivo* experimentation, we could demonstrate that at least some of these host factors provide attractive targets for anti-TB drug development. These results provide a “proof-of-concept” demonstration that targeting host factors subverted by intracellular Mtb provides an attractive and feasible strategy for the development of anti-tuberculosis drugs. Importantly, our findings also emphasize the advantage of such an approach by establishing its equal applicability to infections with Mtb strains exhibiting a range of phenotypic diversifications, including multiple drug-resistance. Thus the host factors identified here may potentially be exploited for the development of anti-tuberculosis drugs.

## Author Summary

The adaptation of *Mycobacterium tuberculosis* (Mtb) involves dynamic interactions with the molecular components of the host cellular machinery. Therefore, targeting relevant host factors may provide an alternate approach for the chemotherapy of tuberculosis (TB). To test this, we first performed an siRNA screen targeting all known kinases and phosphatases in murine macrophages infected with a virulent strain of Mtb. A subsequent validation of this screen then identified several host molecules whose depletion severely affected the intracellular survival of mycobacteria. We also then screened against the identified host targets in cells infected with independent isolates of MDR-Mtb. This exercise identified those host molecules that were indispensable for supporting infection, independent of the phenotypic variations exhibited by the pathogen. Then, by using a pharmacological inhibitor that simultaneously targeted two of these molecules, we were able to demonstrate clearance of both drug-sensitive and drug-resistant strains of Mtb from infected cells. Importantly, this inhibitor was also effective in mice infected with the virulent strain of Mtb. Thus, in addition to demonstrating the feasibility of targeting host molecules involved in supporting intracellular persistence of pathogen for TB therapy, our studies also identify several such molecules that may be exploited for the purposes of drug development.

## Introduction

Successful parasitization of macrophages by *Mycobacterium tuberculosis* (Mtb) reflects the equilibrium between host and pathogen, which is established and maintained through the modulation of macrophage-signaling mechanisms. This leads to the attenuation of several cellular processes that include fusion of phagosomes with lysosomes, antigen presentation, apoptosis, and the bactericidal responses initiated by the macrophage [1], [2], [3]. This attenuation represents the outcome of a dynamic process wherein bacterial molecules interfere with the signaling machinery of the host cell. Although a detailed picture is yet unavailable, many bacterial mediators of virulence have been identified and the strategies employed by them are currently being elucidated [4], [5], [6], [7], [8], [9]. The emerging theme, however, suggests that pathogen-directed manipulation of host processes is achieved through targeted perturbations in the host cell-signaling network [10], [11], [12], [13], [14]. It is these perturbations that then reorient the cellular response to support intracellular survival and growth of Mtb. A better understanding of the mechanisms involved in these perturbations should, therefore, greatly aid current efforts at developing new drugs for tuberculosis (TB).

We undertook this study to identify components of the host cell signaling machinery that are important for regulating an Mtb infection. For this, we performed an siRNA screen against all kinases and phosphatases, in mouse macrophages infected with a virulent strain of Mtb (H37Rv). Such experiments identified several host molecules that were involved either in facilitating, or suppressing, the intracellular infection. By then applying a filter of phenotypic variations, at the level of both drug resistance and intracellular growth properties, we could further distinguish those host molecules that regulated intracellular pathogen load in an Mtb strain-independent manner. A combination of *in vitro* and *in vivo* experimental approaches subsequently enabled us to establish that targeting such host factors indeed provides an attractive alternate strategy for the development of anti-TB drugs. Importantly, our results suggest that such an approach could also potentially address the problem of multiple drug resistance in TB infections.

## Results

### Identifying host proteins that regulate Mtb survival in the macrophage

The siRNA library employed in the primary screen consisted of a pool of two siRNAs per target gene, and the targets included 744 kinases and 288 phosphatases. Cells of the murine macrophage line, J774.1, were first infected with a virulent strain of Mtb (H37Rv), and then transfected with individual target-specific siRNA. The siRNA transfection was performed after infection of cells to ensure an un-hindered uptake of pathogen and, therefore, to select for only those host proteins that were involved in the maintenance of an established infection. The effect of specific silencing on intracellular mycobacterial load was then determined from cell lysates in terms of the colony forming units (CFU) subsequently obtained (Methods).

**Figure 1:**
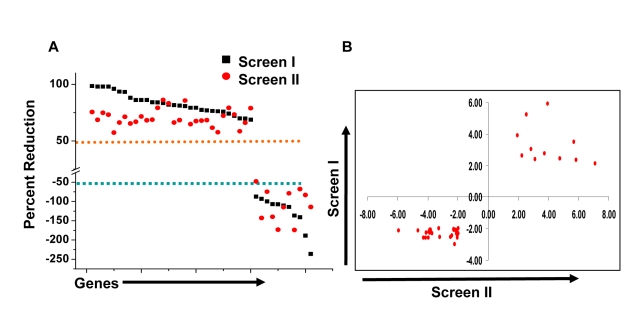
Validation of host-specific proteins involved in regulating Mtb infection. Panel A shows the results obtained for the confirmed targets either in the primary (black squares), or the validation screen (red circles). In view of the inherent differences between the siRNA pools in the two screens, a 50% effect on bacterial load was employed as the cut-off for validation in the latter screen. The scatter plot compares the similarity between the two screens. The Y-axis gives the percent reduction in CFUs obtained relative to the control values (see Text), where a negative value indicates an increase in intracellular bacterial load. These values are the average of 4 different replicates for each screen (Table S1). The two dashed lines in orange and blue indicate the respective cut-off values taken for considering either a decrease, or an increase, in bacterial levels as significant. Panel B shows a scatter plot comparing the fold deviation of the mean values for the individual points in Figure 1A, relative to SD of the mean CFU values for the corresponding controls.

At one level, silencing of several macrophage proteins resulted in a significant decrease in titers of the intracellular bacteria obtained (Table S1). However, there were also many instances where a targeted protein-knockdown led to a marked increase in mycobacterial levels (Table S1). Selection of only those effects as significant, where intracellular bacterial load varied by greater than two standard deviations (2SD) of the mean CFU values obtained for the control wells (Methods), identified 203 target-specific siRNAs (Table S1). To next filter out any non-specificity arising from ‘off-target’ effects of the siRNA, we performed a second screen where the primary ‘hits’ were now silenced with an alternate pool of siRNAs (Methods). Although such an approach likely leads to an increased number of false negatives due to differences in silencing efficiency between the two target-specific siRNA pools, a high degree of confidence is – nonetheless - associated with any reproducible effects that are consequently obtained. This procedure led to a further reduction in the number of significant targets to forty one (Table S1). Then, as the final validation step, we tested whether these target-specific siRNA pools had any effect on the viability of either un-infected, or infected, J774.1 cells over the time course of our experiment (Text S1). In neither of these cases, however, did any of the siRNA pools negatively influence cell viability to an extent that was greater than 1.5 SD of the mean values obtained for the control wells (Table S2). Therefore, these forty one targets identified by the corresponding set of siRNA pools were taken as the validated target group. The effect of silencing of each of these on the intracellular bacterial load is shown in Figure 1A, whereas Figure 1B confirms that the siRNA pools used in the primary and in the validation exercise both yielded effects that were greater than 2SD of the mean CFU value obtained for the corresponding control wells. As is evident, the effects of target-suppression ranged from a near complete elimination of the infection, to a significant enhancement in intracellular bacterial titers (Fig. 1A). Thus, adaptation of mycobacteria in the intracellular milieu likely involves both facilitating and inhibitory contributions from some of the components of the host cell signaling machinery. The validated targets are listed in Table 1, which also includes a gene ontology-based classification of their functional roles. A more detailed description of these genes and their known association with disease and other cellular functions is provided in Table S3.

**Table 1:** Functional Classes of ‘Hits’.

Gene Accession	GeneSymbol	GO Classes
NM_007377	Aatk	Integral to membrane, Protein serine/threonine kinase activity
*NM_009594*	*Abl1*	*Actin cytoskeletal organization, Magnesium ion binding*
NM_133770	ADCK4	Protein serine/threonine kinase activity, Transferase acivity
NM_134079	Adk	dATP biosynthetic process, Transferase activity
NM_130863	Adrbk1	G-PCR tyrosine kinase activity, Protein serine/threonine kinase activity
NM_015804	Atp11a	Phospholipid transport, ATP biosynthetic process
NM_029097	Atp13a2	ATP biosynthetic process, Cation Transport
NM_144921	Atp1a3	Ion Transport, ATP Biosynthesis
NM_175025	Atp2c1	Calcium ion transport, Golgi Apparatus
NM_013482	BTK	I-Kappab kinase/ NFKappab cascade, Cytoplasmic Vesicle
NM_001025438	CaMK2d	Calmodulin Binding, Calcium Ion Transport
*NM_007631*	*Ccnd1*	*Endoplasmic reticulum unfolded protein response, Wnt Signaling*
NM_007658	Cdc25a	Hydrolase activity, Protein tyrosine phosphatase activity
NM_023117	Cdc25b	Cytoskeleton, Protein tyrosine phosphatase activity
NM_009860	Cdc25c	Hydrolase activity, Protein tyrosine phosphatase activity
NM_194444	CDK10	ATP binding, Transferase Activity
*NM_007691*	*Chek1*	*Cytoskeleton, Protein serine/threonine kinase activity*
NM_027874	Csnk1d	Wnt receptor signaling pathway, Protein serine/threonine kinase activity
NM_013767	Csnk1e	Wnt receptor signaling pathway, Protein serine/threonine kinase activity
NM_007828	DAPK3	Induction of apoptosis, Protein serine/threonine kinase activity
*NM_138306*	*Dgkz*	*Diacylglycerol Kinase activity, Negetive regulation of Ras protein signal transduction*
NM_023173	Dusp12	Protein tyrosine/serine/threonine phosphatase activity, Metal ion binding
NM_019819	Dusp14	Protein tyrosine/serine/threonine phosphatase activity, Hydrolase Activity
NM_026268	Dusp6	Protein tyrosine/serine/threonine phosphatase activity, Hydrolase Activity
NM_178676	Entpd3	Hydrolase Activity, 5′- Nucleotidase Activity
NM_008363	IRAK1	TLR2/4 signaling pathway, Response to LPS, Peptidoglycan
NM_181593	Itpkc	Calmodulin Binding, Inositol triphosphate 3-Kinase activity
NM_025730	LRRK2	Memberane Raft, Trans golgi network
*NM_011948*	*MAP3K4*	*Map kinase kinase kinase activity, Metal ion binding*
NM_008825	Pfkfb2	Fructose Metabolic Process, Hydrolase activity
*XM_134059*	*Ppapdc1*	*Hydrolase activity, Integral to membrane*
NM_011100	Prkacb	G-protein signaling, Coupled to cAMP nucleotide second messenger, Transferase activity
NM_153744	Prkag3	Lipid Biosynthetic Process, Glycogen Biosynthetic Process
NM_016795	SRPK1	ATP binding, Protein serine/threonine kinase activity
NM_009370	TGFβR1	Protein Serine/ Threonine Kinase Activity, TGFb Receptor Activity
*NM_011587*	*TIE1*	*Receptor Activity, ATP binding*
*NM_021450*	*Trpm7*	*Calcium ion transport, Actin Binding*
NM_183099	TSSK5	Protein Serine/Threonine Kinase Activty, Metal Ion Binding
*NM_026765*	*Uckl1*	*Metabolic Process, Phospho Transferase Activity*
*NM_009469*	*ULK1*	*Regulation of autophagy, Autophagic Vacuole*
*NM_009516*	*Wee1*	*Protein tyrosine/serine/threonine kinase activity, Metal Ion Binding*

**Figure 2:**
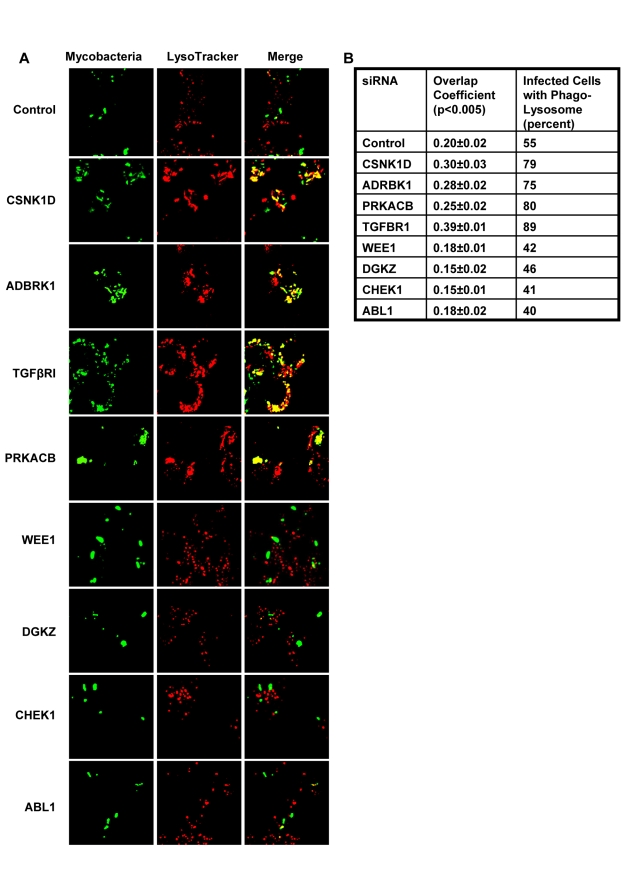
Alterations in levels of host-specific proteins influence the fate of intracellular mycobacteria. Figure shows the effect of the indicated target-specific siRNAs, or of non-silencing siRNA (GFP-specific, Control), on the extent of co-localization of mycobacteria with acidified lysosomes. Mycobacteria were labeled with the lipophilic dye PKH67 (green) prior to infection. Infected cells were then monitored at 72 hours by confocal laser scanning microscopy to determine the extent of co-localization of mycobacteria with acidified lysosomes (panel A), and the percent of cells in which such a co-localization was visible (panel B). Acidified lysosomes were stained with Lysotracker (red) (see Methods and Text S1). In panel A, the yellow color in the ‘merge’ signifies co-localization of the two. The numbers in panel B give both the overlap coefficient of the bacteria with the lysotracker, as well as the percent of cells in which this co-localization was detected. More than 40 infected cells (i.e. positive for fluorescent mycobacteria) were examined in slides generated from three separate experiments and the values in panel B represent the mean ±S.D. Further, the values for both parameters were found to be significantly different at p<0.005. As is evident, both the overlap coefficient and the fraction of cells with mycobacteria in acidified lysosomes were higher than control values following depletion either of *ADRBK1*, *CSNK1d*, *TGF*β*RI*, or *PRKACB*. In contrast, these values were low when *ABL1*, *CHEK1*, *DGKZ* or *WEE1* was depleted. This correlates well with the corresponding results obtained in terms of the CFU values shown in Figure 1. The extent of siRNA-mediated silencing obtained for each of these target proteins is shown in Figure S3.

We next took four representative examples from each of the two distinct groups in Figure 1, and examined – by confocal microscopy - whether the observed modulation in CFU values also correlated with alterations in the level of mycobacteria localization, within the lysosome of the host cell. Interestingly, in infected cells treated with non-silencing siRNA, the co-localization of H37Rv with acidified lysosomes could be detected – with a mean overlap coefficient of 0.20 - in only a little over 50% of the cells, (Fig. 2). In contrast, those instances where silencing yielded a reduced CFU count in Figure 1 (*Csnk1d*, *Adrbk1*, *Prkacb*, *and TgfβrI*), showed a marked increase in mycobacterial co-localization with acidified lysozomes, both at the level of individual cells, as well as at that of the proportion of cells showing such co-localization (Fig. 2). On the other hand, values for both of these parameters were significantly reduced for the cases (*Wee1*, *Abl1*, *Dgkz*, *and Chek1*) where target gene silencing resulted in an increase in the CFU counts (Fig. 2, compare with Fig. 1).

Thus, at least for the representative cases shown in Figure 2, the observed alterations in the CFU counts constitute a true reflection of shifts in the fate of the intracellular bacteria. Finally, an examination of the gene expression profiles both in un-infected J774.1 cells, and in cells infected with H37Rv either for 16h, 48h, or 96h, confirmed that the genes coding for all of the proteins listed in Figure 1 were indeed expressed in uninfected cells, albeit to different levels. Further, expression levels of thirty-four of these remained largely unaffected following infection of the cells (Table S4).

### Identification of common host-derived protein targets for MDR-Mtb

**Figure 3:**
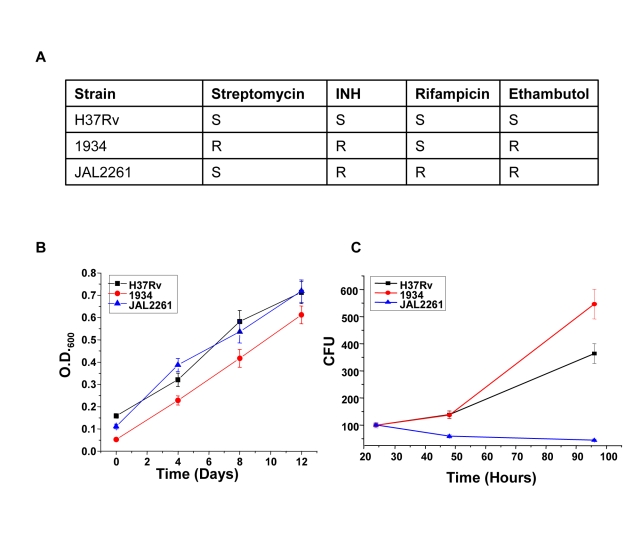
Intracellular growth properties of H37Rv and the two MDR isolates. Panel A depicts the sensitivity profile of the clinical isolates against the four standard, first-line, anti-mycobacterial drugs (S, sensitive; R, resistant). Panels B and C show the growth properties of H37Rv, and the two clinical strains either in extracellular cultures (panel B), or in infected J774.1 cells (Panel C). For the former, mycobacteria were seeded in culture medium at 2×10^7^/ml and the growth monitored by measuring absorbance at 600 nm. Values obtained in the log phase of growth are shown here. For Panel C, cells were separately infected with each of the three strains and CFU counts determined in lysates obtained at 24, 48, and 96 hrs later (Methods). A parallel set of infected cells was also taken for determining the cell viability, by an MTT assay, at each of these time points. Values presented are the mean (± S.D.) of triplicate measurements of CFU counts, as a function of the absorbance obtained in the MTT assay at each of the time points. The corresponding growth rate of these strains in primary mouse macrophages is shown in Figure S1.

We next asked whether the proteins identified against H37Rv in Figure 1 would also be relevant for infections with field isolates of Mtb, particularly for those exhibiting multiple drug resistance (MDR). To address this we took two independent clinical isolates of MDR-Mtb, each displaying a distinct drug-sensitivity profile (Fig. 3A). The relative growth rates of these two isolates with, that of H37Rv, was first compared both in extracellular cultures, as well as in infected cells. Interestingly, while all the three isolates displayed similar growth properties under extracellular conditions (Fig. 3B) these, however, differed markedly when measured within infected J774.1 cells (Fig. 3C). In this latter experiment, a progressive increase in CFU counts was obtained for H37Rv over the time course studied whereas the MDR strain 1934 displayed an accelerated growth rate between the 48h and the 90h time points. In stark contrast, however, virtually no increase in bacterial titers could be observed for the other MDR strain, JAL2261, at any of the time points studied. A reduced intracellular growth rate for JAL2261, in comparison with the other two strains, was also similarly observed in primary murine macrophages (Fig. S1). Thus the three Mtb strains studied exhibit diverse growth properties within J774.1 cells.

**Figure 4:**
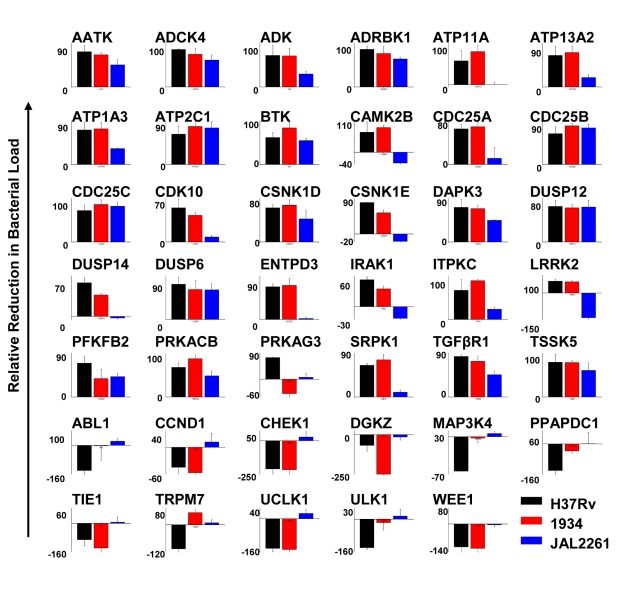
Overlapping sensitivities of different MDR-Mtb strains to the effects of target-specific siRNA. Cells infected with either 1934, or with JAL 2261, were screened against the target-specific siRNAs shown in Figure 1. The resulting CFUs obtained are again presented in terms of the percent reduction obtained relative to control values, where a negative value indicates an increase in bacterial titers. The corresponding values obtained for H37Rv are also included for the sake of comparison, and the color code used for the individual strains is indicated. Values are the mean (± S.D) of the total of four values obtained from two separate experiments (i.e. CFUs from two lysate dilutions per experiment).

We next infected J774.1 cells with each of the MDR-Mtb strains and tested for the effects of treatment with the siRNAs described in Figure 1. The results obtained are shown in Figure 4. While the overall profiles obtained for both 1934 and JAL2261 show distinctions from that for H37Rv, some overlaps were – nonetheless – clearly evident. This was particularly true in the case of 1934 where, of the 41 targets tested, comparable effects of target silencing (i.e. within a 30% deviation) on both 1934 and H37Rv were observed in 31 cases (Fig. 4). The majority of these instances (25 of 31) represented the group wherein siRNA treatment yielded a reduction in the intracellular bacterial load, whereas a relatively poorer correspondence was obtained for the group where increased bacterial titers were observed (6 out of 11; Fig. 4). Discrepant results were obtained for *Trpm7* and *Prkag3* although the cause for this is presently unclear.

In contrast to 1934, a comparison between the results obtained for JAL2261 and H37Rv revealed a significantly reduced overlap, with the similarity in effects being restricted to only 11 instances of siRNA treatment (Fig. 4). Further, all of these cases derived exclusively from the subset involving a consequent reduction in the level of the intracellular mycobacteria (Fig. 4). This reduced sensitivity of JAL2261, to perturbations in host protein levels, is probably consistent with the fact that it exists in a non-dividing, or slow-dividing, state within the host cells. Thus the results in Figure 4 identify 11 host targets, whose depletion uniformly led to a reduction in intracellular load of all the three Mtb strains tested.

### Host factors regulating intracellular Mtb levels provide attractive targets for drug development

**Figure 5:**
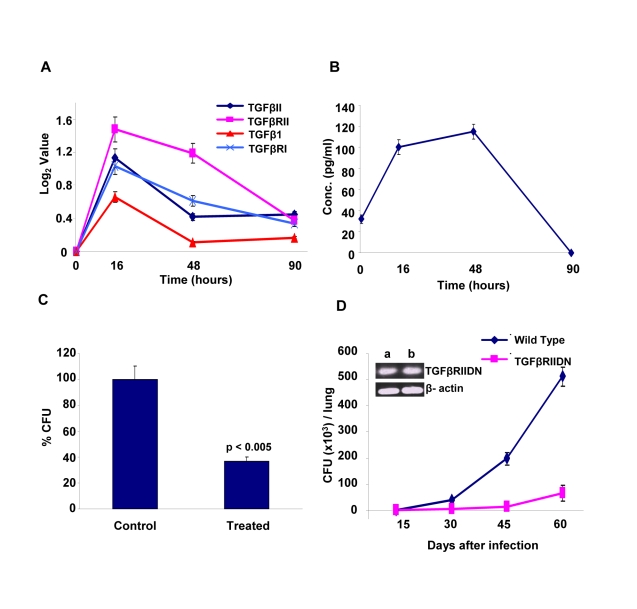
Mtb infection regulates levels of TGFβ and its receptor in host cells. Panel A depicts the time-dependent changes in expression levels of the cytokines *TGFβ1*, *TGFβII*, and their receptors *TGFβRI* and *TGFβRII* in J774.1 cells infected with H37Rv (see Methods and Text S1). Values were taken from the results from our gene expression analysis described in Table S4. Panel B describes the levels of TGFβI protein in the culture supernatants of J774.1 cells infected for the indicated times with H37Rv. The concentration of this cytokine was determined using the TGFβI Lincoplex kit (Text S1), and values are the mean ± S.D. of three separate experiments. Panel C shows the results of an experiment where H37Rv-infected J774 cells were cultured either in the presence (Treated) or absence (Control) of neutralizing monoclonal antibodies against TGFβ (clone 1D11). This antibody neutralizes all the three isoforms of TGFβ. The antibody was first added (final concentration of 0.5 µg/ml) at 12h after infection and then replenished at every 12h thereafter, up to 72 h post-infection. At 90h post-infection cells were lysed and bacterial loads determined in terms of the CFU values (mean ± S.D. of three experiments). Statistical significance of the difference seen is also indicated. For the experiment in Panel D, either wild type (Balb/C) or TGFβRIIDN (Balb/c background) mice were infected with H37Rv through the aerosol route as described in Methods. These mice were then sacrificed at the indicated time points and mycobacterial CFUs present in the lung homogenates was determined. Values obtained at time point are the mean (± S.D.) of that obtained in four mice. Results shown here are from one of three separate experiments taking four mice/group in each. For the remaining two experiments, CFU values obtained at day 60 for the wild type versus the TGFβRIIDN mice were 5.7±1.2×10^5^ versus 0.42±0.1×10^5^ and 2.8±0.4×10^5^ versus 0.1±0.06×10^5^. The inset in the panel compares mRNA levels of TGFβRIIDN in peritoneal macrophages (a) with that in splenic lymphocytes (b), against actin as the control for semi-quantitative comparison.

The identification of host factors commonly implicated in regulating intracellular levels of all the three Mtb strains tested raised the possibility that at least some of these may serve as targets for the development of drugs against both drug-sensitive and drug-resistant forms of Mtb infection. At least at the level of ‘proof of principle’, employing existing chemical inhibitors of any of these targets can readily test such a possibility. Therefore, for the present purposes, we chose to examine the effects of inhibition of TGF-β type-1 receptor (TGFβRI). Our choice of this target was based on the results of our microarray experiments which indicated that transcript levels of both this receptor, as well as that of its isoform TGF-β type-2 receptor (TGFβRII) were significantly increased in cells infected with Mtb for 16h, although these levels then returned to their basal values at the later times (Fig. 5A). Further, expression of the genes for the cognate ligands, TGFβI and TGFβII were also induced (Fig. 5A). Here, although the extent of induction of TGFβI was not significant (i.e. below the cut-off threshold) this, nonetheless, translated into a significant increase in the levels of this cytokine in the culture supernatants (Fig. 5B). It was, therefore, logical to infer that secretion of TGFβ by infected cells would lead to the corresponding activation of its receptor, either through autocrine or paracrine mechanisms. Further, at least based on the results of our siRNA screen, it seemed likely that this activation was somehow relevant for maintenance of the intracellular pathogen.

To experimentally verify the inferred role for TGFβ-dependent activation of its receptor, we added neutralizing anti-TGFβ antibodies to cultures of H37Rv-infected J774 cells and then determined its consequent effect in terms of the mycobacterial CFUs obtained. Figure 5C shows that addition of anti-TGFβ antibodies resulted in a substantial reduction in intracellular Mtb load, thus confirming the relevance of this cytokine in regulating intracellular Mtb. To further establish this, we also performed experiments in mice that were transgenic for the dominant negative form of the TGFβ receptor type II (TGFβRIIDN). Although our screen identified TGFβ receptor type-I as the hit, signaling through TGFβ1 involves the formation of an active heteromeric complex between the type-I and type-II receptors. Thus, binding of TGFβ to the type-II receptor leads to the recruitment and phosphorylation of the type-I receptor, with the subsequent activation of downstream pathways [15]. Consequently, TGFβ-dependent signaling would be similarly affected by either silencing the type-1 receptor, or over–expressing the dominant negative form of the type-II receptor. As shown in Figure 5D, TGFβRIIDN mice were significantly more resistant to an aerosol route of infection with H37Rv, than their wild type counterparts. This was consistent with the fact that macrophages isolated from the transgenic mice indeed showed over-expression of TGFβRIIDN (Fig. 5D). Collectively then, the results in Figure 5 provide strong experimental support for the likelihood that TGFβ-dependent activation of its receptor on macrophages is critical for the survival of intracellular Mtb.

To inhibit TGFβRI activation, we employed the compound 4-[4-(2,3-Dihydro-1,4-benzodioxin-6-yl)-5-(2-pyridinyl)-1H-imidiazol-2-yl]benzene (D4476). Our choice of this inhibitor was guided by the fact that, in addition to TGFβRI, this compound is also known to inhibit casein kinase 1 (CSNK1) [16], [17], another member of our validated target list in Figure 4. Interestingly, CSNK1 also represents a downstream intermediate in the TGFβR signaling pathway, and has been shown to regulate both ligand-independent (i.e. basal), as well as ligand-induced signaling processes [18]. Thus, it was anticipated that the simultaneous inhibition of TGFβRI and one of its downstream signaling intermediates would yield a more effective inhibition of TGFβ-dependent signaling and, therefore, a more potent effect on intracellular Mtb levels.

**Figure 6:**
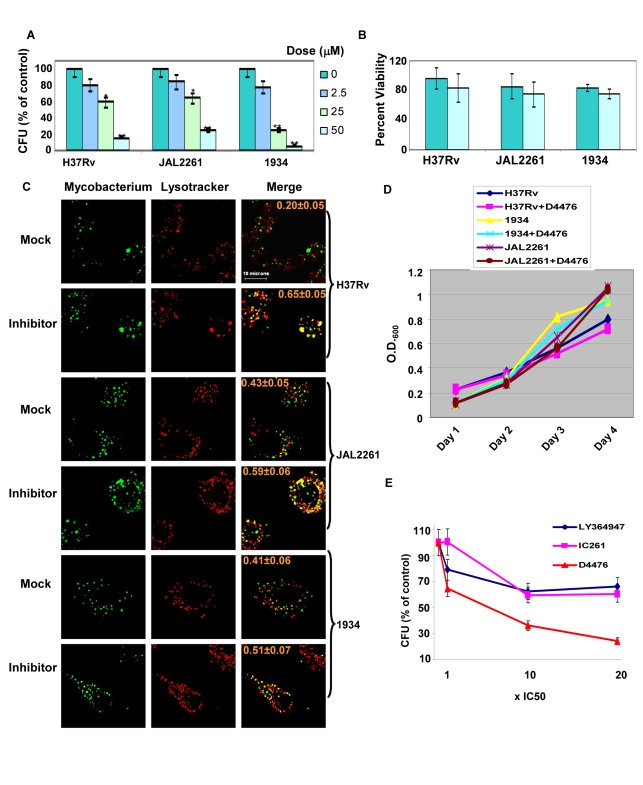
Dual inhibition of TGFβR1 and Casein kinase eliminates Mtb from infected cells. J774.1 cells independently infected with each of the three isolates were treated with either the vehicle only or with the indicated concentrations of D4476 (Dose). Addition was performed at 16h and the medium containing the appropriate dose of inhibitor was refreshed every 24 h up to the 64 h time point. At the end of a total culture period of 90h, cells were lysed and CFUs determined as described in Methods. Panel A shows the results where the CFU values from inhibitor-treated cells are expressed as a percent of that obtained from correspondingly infected cells treated with vehicle only. Panel B shows the viability of infected J774.1 cells obtained at the 90h time point either in the presence (light blue bars), or absence (light green bars) of 50µM D4476. In both cases, values are the mean (± S.D.) of three separate determinations. The p-values are indicated by the stars; *<0.05 and **<0.01. Panel C shows the results of a parallel experiment where the extent of co-localization of PHK67-stained mycobacteria with acidified lysosomes (stained with Lysotracker) was determined at 72h as described for Figure 2. For each of the isolates (indicated on the right) the results for treatment of infected cells either with vehicle only (Mock), or with D4476 (50 µM, Inhibitor) is shown (indicated on the left side of the panel). Images shown for each of these groups are those obtained for PHK67-labeled Mtb (Mycobacterium), acidified lysosomes (Lysotracker), or a merge of the two (Merge). The numbers indicate overlap coefficient of the bacteria with the lysotracker and were obtained as described for Figure 2. Panel D depicts the growth profiles of the three Mtb isolates obtained either in the absence, or presence of 50 µM D4476. At a final concentration of 10 µM, D4476 inhibits the activities of purified CSNK1 and TGFβRI by >90 and 78% respectively [16]. Panel E compares the efficacies of the TGFβR and CSNK1 dual inhibitior D4476 with that of either the TGFβR-specific inhibitor LY364947, or the CSNK1-specific inhibitor IC261. The doses used for each inhibitor was in terms of multiples of their respective IC_50_ values as indicated, and the procedure employed was identical to that described for Panel A. Values are the mean (± S.D.) of three separate determinations.

Cells infected either with H37Rv, 1934, or JAL2261 were treated with increasing doses of D4476, and the consequent effect on intracellular pathogen load was then determined as the resulting CFU values obtained (Fig. 6A). A dose-dependent decrease in mycobacterial CFUs, with increasing inhibitor concentrations, was clearly obtained for all the three isolates tested (Fig. 6A). Importantly, this effect was specific for the mycobacteria and this drug displayed no toxicity towards the host cell (Fig. 6B). In parallel studies employing confocal microscopy we observed that treatment with the inhibitor also led to a corresponding increase in localization of each of the mycobacterial isolates within acidified lysosomes of the cell (Fig. 6C). This further supports that treatment with the inhibitor leads to an enhanced clearance of the intracellular pathogen. Importantly, the effect of D4476 addition was specific for intracellular mycobacteria, with no significant effect on mycobacterial growth in extracellular cultures (Fig. 6D). This confirms that the inhibitor did not directly target Mtb. Rather it more likely interfered with the intracellular survival mechanisms of the pathogen. Importantly, from the standpoint of pharmacological intervention, that simultaneous inhibition of TGFβRI and its downstream signaling intermediate leads to a more potent effect on intracellular Mtb could be demonstrated by comparing the relative efficacy of D4476 with that of inhibitors specific for only either TGFβRI (LY364947 [19]) or CSNK1 (IC261, [17]) (Fig. 6E).

The inhibitory effect of D4476 on intracellular Mtb survival could also be demonstrated in primary murine macrophages infected with each of the three Mtb strains. Thus, consistent with the findings in J774 cells, addition of D4476 to infected primary macrophages resulted in enhanced co-localization of all three mycobacterial strains with the lysosome, and also a significant reduction in CFU count in each of these cases (Figure S1). Thus the cumulative results in Figure 6 confirm a key role for TGFβR signaling in regulating intracellular Mtb survival, but in a manner that is independent of at least the spectrum of phenotypic variations encompassed by this group of isolates.

### Compound D4476 also eliminates Mtb from infected mice

**Figure 7:**
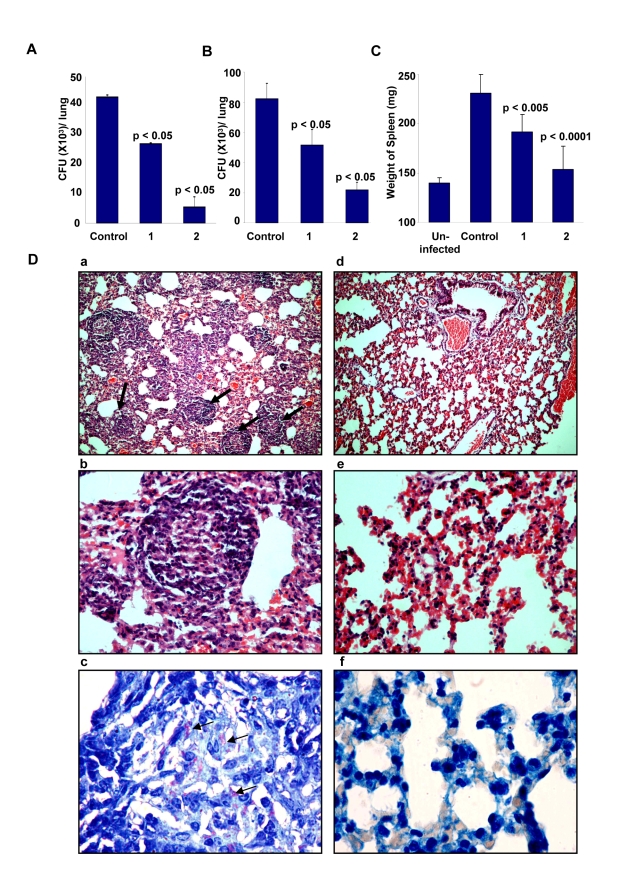
Efficacy of D4476 in the murine model of Mtb infection. Groups of naive mice (Female BALB/c mice 4–6 wk of age at 4/group) were infected with 1×10^6^ *M. tuberculosis* H37Rv via the tail vein. One group of mice was sacrificed 24 h later and lung homogenates were plated onto 7H11 agar plates for confirming infection. At ten days post infection, compound D4476 at either 2 nmol/g (1) or 4 nmol/g of body weight (2) was injected into the tail vein of mice for treatment. The *i.v.* injection was repeated on seventh day of treatment. In addition the inhibitor was also given intraperitoneally on the third, fifth, tenth and twelfth day after initiation of treatment. Here, a parallel group of mice received an identical treatment regimen with the vehicle only (Control). At fourteen days following treatment, mice were sacrificed and lung enriched using a homogenizer. An aliquot of the homogenate was lysed and plated onto 7H11 agar plates in serial dilutions for determining the mycobacterial load. The results are shown as mean (± SD) CFU values in panel A. This experiment was also repeated with the higher dose of D4476 (i.e. with 4 nmol/g of body weight with 4 mice/group). Here, the mean CFU values (± SD) obtained for the Control (vehicle only) and the D4476-treated groups were 68±1.4×10^3^ and 8.0±1.8×10^3^ respectively. Panel B shows the results of a similar experiment performed in mice that were infected with H37Rv through the aerosol route as described in Methods. Here 8 mice were used in each group and the values (mean ± S.D.) obtained in lung homogenates at 5 weeks after infection are shown. Here again the control group was that which received the vehicle only (i.e. DMSO) in parallel with D4476 administration in the other groups. Shown in Panel C are the mean (± S.D.) weight of the spleens obtained from either uninfected mice (Uninfected), infected mice without any inhibitor treatment (Control), or infected mice treated with either 2 (1) or 4 (2) nmol/g of body weight of D4476. These spleens are from the experiment described in Panel B. For panels A to C the p values for individual differences are indicated. Panel D shows lung sections of infected mice stained with hematoxylin / eosin 24 days after infection at ×10 (a) and ×40 (b). The typical pathology associated with experimental tuberculosis infection in mice is seen. Multiple well-formed epithelioid cell granulomas with mantle of lymphocytes are seen (indicated by the arrows). In contrast, mice treated with D4476 (160µg/kg body weight) maintained almost normal lung architecture ×10 (d), ×40 (e) with non-specific inflammation, and no granuloma formation was observed in the lungs of these mice. Further, untreated mice reveal large number of acid-fast bacilli when visualized at 100× (c) (indicated by the arrows); whereas very few bacilli were observed in mice treated with D4476 (4 nmol/g body weight) (f).

To further validate the relevance of targeting host factors, we also examined the efficacy of D4476 in the murine model of TB infection. For this, BALB/c mice were intravenously infected with H37Rv and these mice were then treated with two different concentrations of D4476. Treatment was initiated at ten days after the infection, and included six administrations of the relevant dose of the inhibitor (Text S1). As shown in Figure 7A, a clear reduction in CFU counts was obtained from the lungs of infected mice treated with D4476. Further, the magnitude of this inhibition was sensitive to the dose of the compound with a dose of 4 nmol/g of body weight yielding a nearly 80% reduction in CFU counts. Similar results were also obtained in mice infected through the aerosol route (Fig. 7B), confirming that the efficacy of D4476 was independent of the route of infection employed. D4476-dependent clearance of infection was also evidenced through recovery from the infection-induced splenomegaly (Fig. 7C). Further, histochemical staining of lung sections also revealed a significant reduction in the number of epitheloid cell granulomas, and in that of acid-fast staining bacilli, in treated versus the untreated mice (Figure 7D).

The collective results in Figure 7, therefore, demonstrate the *in vivo* efficacy of D4476 in terms of eliminating an Mtb infection. While its potency may be considered to be low we emphasize, however, that our objective here was not to propose this compound as a drug against TB. Rather, these and the preceding experiments were merely intended to reveal and highlight the feasibility of targeting relevant host factors, as an alternate strategy for the chemotherapy of TB.

## Discussion

Recent years have witnessed a resurgence of efforts directed at tuberculosis (TB) drug research and development. This has been spurred by the increasing incidence of drug resistance in the infected population, engendering an urgent need for the development of improved regimens for TB treatment. Although drug resistant - particularly multi- and extremely-drug resistant - TB poses a daunting challenge, the situation is further complicated by the predominance of latent and/or persistent forms of TB infection in the population [20]. These latter infections are characterized by phenotypic resistance - or tolerance - to drugs, despite being genotypically sensitive to them [20]. Indeed, latent/persistent infection constitutes over ninety nine percent of the TB infected population worldwide [20].

Conventional approaches to drug research have targeted unique processes or enzymes of the pathogen. However, despite its many successes, this approach suffers from the risk of generating newer variants exhibiting drug resistance [21]. Further, this strategy is also limited by its inability to address infection states where the pathogen is metabolically less active such as in persistent or latent infections. This issue is especially relevant in the case of Mtb infections. An alternate paradigm for drug discovery has recently emerged, at least for intracellular pathogens [22]. This is based on the fact that the survival of an intracellular pathogen in the hostile cellular environment requires it to exploit and subvert various host factors. Therefore, identifying - and then targeting – such host factors should provide an additional route for the therapeutic management of these infectious diseases. Here, the anticipations also are that such a strategy will be less likely to induce microbial resistance [22].

To explore the possibilities offered by this avenue, siRNA-based screens are now being widely employed to identify such host factors for a range of viral, bacterial, and parasitic infections [23], [24], [25], [26], [27], [28], [29]. In this connection, a recent screen of the human kinome identified a kinase cluster around protein kinase B (PKB) as being obligatory for the intracellular survival of *Salmonella typhimurium* [30]. Interestingly, while inhibitors of PKB were effective against this pathogen, they were also capable of partially inhibiting infection of human macrophages with a strain of MDR-Mtb. We, therefore, undertook the present study to further explore the potential of such an approach in the specific context of Mtb.

Our siRNA screen successfully identified several host factors involved in the regulation of H37Rv survival within the milieu of the host macrophage. Interestingly, this group of target proteins included those that facilitated, as well as those that impeded Mtb survival. While a mechanistic resolution of the biochemical pathways involved is clearly needed, this identification of molecular components with opposing functional roles strongly supports the existence of a host-specified axis that regulates the fate of intracellular Mtb. Such an axis probably reflects the level of equilibrium achieved between the intracellular processes initiated to eliminate the infection, and the pathogen-mediated manipulation of the host machinery in its own favor [31]. In this connection, it is pertinent to note that previous studies have identified several host proteins that play an important role during the course of an Mtb infection. However, with the exception of a few examples [32], the majority of these proteins are involved in processes that occur either during, or soon after, the endocytic uptake of Mtb. Thus, for example, the role of the calcium signaling pathway- involving PI3K, PKB, CaMKII and Coronin 1 – in regulating endocytosis and subsequent fusion of phagosomes with lysosomes has been well studied [1], [12]. Similarly, there is also information available on the biochemical pathways that mediate microbicidal responses of the infected macrophage [33]. In contrast to these early responses, however, our screen was designed to specifically capture those host molecular components that are involved in the later time window of the infection process. That is, in the window where the pathogen is either in the process of establishing, or has established, a dynamic equilibrium with the intracellular machinery of the host cell. Consequently, our present study examined a relatively less explored facet of macrophage infection by Mtb and, therefore, identified several novel host molecules whose role in regulating intracellular Mtb has not been hitherto suspected. From the standpoint of targeting host factors as a drug development strategy for TB, we believe that it is such factors that regulate the maintenance of an established infection that would be more relevant.

An important aspect of our studies was the additional filtration, of the identified H37Rv-specific host factors, against MDR-Mtb variants that not only exhibited altered drug-sensitivity profiles, but also altered properties of intracellular growth. Interestingly, a significant proportion of these factors could be validated against the other rapidly growing strain, 1934. In contrast, only a small subset of these targets retained efficacy against JAL2261, a strain whose levels did not significantly increase within infected cells. These differences are consistent with at least *a priori* expectations that the extent of cross-regulatory interactions between host and pathogen would correlate directly with state of replication activity of the pathogen.

The net outcome of our experiments was the identification of a core list of host targets that were involved in regulating survival of Mtb, independent of variations in either drug sensitivity profiles or growth properties in the host cell. Further, we could also demonstrate, at the level of ‘proof of principle’, that at least some of these host factors may provide novel targets for the development of anti-TB drugs. This was exemplified by our findings that the simultaneous inhibition of TGFβRI and CSNK1 substantially inhibited intracellular survival of both drug-sensitive and multiple drug-resistant strains of MTB. In addition to J774.1 cells, this effect was also observed in primary murine macrophages. More importantly though, our experiments involving D4476 administration to H37Rv-infected mice also provided *in vivo* validation for the possibility of targeting host factors as a possible approach for TB therapy. Here, it will be important to test the potential applicability of the remaining host factors identified by our screen in this regard. Further, the mechanism by which inhibition of TGFβRI and CSNK1 induces elimination of the infection is also of interest.

Thus, in summary, our present results highlight the existence of host factors that regulate the intracellular survival of Mtb in a manner that is insensitive to variations in either the drug-sensitivity profile, or the intracellular growth properties. In addition, we also provide proof-of-concept demonstration that targeting at least some of these molecules can provide an alternate approach for the chemotherapy of TB. Again as demonstrated here, a highlight of such a strategy would be that it holds the promise of eventually being able to develop suitable drugs that function in a manner that is independent of the phenotypic and genotypic diversification exhibited by Mtb in the field [20]. However, further validation in the context of human infections will be necessary before such a promise can be realized. Further, the issue of possible toxicity to the host cell - as a result of inhibition of key host molecules - may also require to be addressed.

## Methods

A detailed description of all the experimental protocols employed, and the standardization procedures are provided in the *Text S1*.

### Ethics statement

All animal experiments were carried out in accordance with guidelines approved and created by the ICGEB animal ethics committee.

### Animals

Female BALB/c mice 4–6 wk of age kept in pathogen free environment. TGFβRDN (TGFβ receptor dominant negative, BALB/C background) mice, 6 to 8 weeks of age, were purchased from Jackson Laboratory, Bar Harbor, Maine. These mice were maintained and breed in a specific-pathogen-free biosafety level-3 facility.

### Cells and culture

Murine macrophage cell line J774.1 (American Type Culture Collection) was used in this study. J774.1 cells were cultured in RPMI 1640 (Gibco Laboratories) supplemented with 10% FCS (Hyclone) and were maintained between 2 and 10×10^5^ cells per mL at 37°C in a humidified, 5% CO_2_ atmosphere. Before infection cells were plated in 96 well plates at 2.5×10^4^ cells per well overnight.

### siRNA library

Mouse Phosphatase siRNA set V 1.0 and Mouse kinase siRNA set V 1.0 library from Qiagen (two siRNAs/target) were used for the study. For validation experiments siRNAs against the kinases were obtained from Sigma siRNA (MISSION siRNA Mouse Kinase Panel, three siRNAs/target), whereas the phosphatase-specific siRNAs were procured from Dharmacon (SMARTpool) (four siRNAs/target).

### Infection of cells and the siRNA screen

J774.1 cells (25,000 cells/well in 96-well plates) were infected with mycobacteria at an MOI ∼10 (10 bacteria /cell, Figure S2). After 4h, infected cells were washed twice with warm RPMI and treated with gentamicin (100 µg/ml) for 2h to remove any remaining extra cellular bacteria, and then in complete RPMI containing 10 µg/mL gentamicin for the rest of the experiment. These cells were then transfected with siRNA at a final concentration of 100nM using hiperfect transfection reagent (Qiagen) according to manufacturer's protocol. At 48h a second siRNA treatment was performed (see *Text S1*), and the cells cultured for an additional 36h (i.e. a total culture period of 90h after initiation of infection). At this point cells were solubilized in 50 µL of 0.06% SDS (in 7H9 medium), and CFUs determined using either the undiluted lysate, or from lysate dilutions of 1∶10, or 1∶100. Each siRNA pool was evaluated in duplicate and the mean of the CFU values, obtained at the two cell lysate dilutions was determined. Each 96-well plate also included six negative control wells. In two of these cells were treated with scrambled siRNA following infection, whereas another two included infected cells that were transfected with GFP-specific siRNA. In the remaining two wells, infected cells were treated only with the transfection reagent (Hiperfect). The mean CFU count obtained from all of these six wells, in each 96-well plate, was taken as the control value for that plate for determining the effects of target-specific siRNAs. The standard deviation (SD) for the control values was also calculated. A cut off of two SD from the mean value of control wells was employed to designate the effects of a given siRNA treatment as significant. The ‘hits’ obtained from this primary screen were then further validated with siRNA pools obtained from alternate sources as described above. Here, ‘hits’ were confirmed on the basis of the percent change in CFUs from the mean control value, in addition to an SD cut off value of two. That is, an increase or decrease of 50% from the control value for an siRNA with the additional caveat that this deviation from the mean control value was greater than 2SD was selected as validated (see Fig. 1). The validation exercise involved two separate experiments, whereas the subsequent comparison of the effects of the validated siRNA pools in cells infected with H37Rv, with that in cells infected with 1934 and JAL2261 was also ascertained in two additional independent experiments.

### RNA isolation and microarray analysis

In two separate experiments, cells were plated in six-well plate (2×10^6^ cells per well) and infected with H37Rv as described above. At 16h, 48h, and 96h later these, and uninfected, cells were lysed and RNA was isolated with trizol. One-color microarray-based gene expression analysis was performed by hybridizing against a mouse whole genome array consisting of probes for 44,000 genes (Agilent).

### Confocal microscopy

Bacteria were stained using the membrane stain PKH67 (Sigma) according to the manufacturer's protocol. J774.1 cells were seeded onto #1 thickness, 12 mm diameter glass cover-slips pre-coated with fibronectin in 24-well tissue culture plates at a density of 0.07×10^6^ cells per cover-slip, respectively and infected with stained bacteria using the protocol as described above. Cells were incubated with 100nM Lysotracker during the last hour of the 72 hr chase at 37°C and then fixed with 4% para-formaldehyde (Sigma). The cover slips were washed thoroughly with PBS and were mounted on slides with Antifade (Biorad). Stained cells were observed with a Nikon TE 2000E laser scanning confocal microscope equipped with 60×/1.4 NA PlanApochromat DIC objective lens, and the extent of bacterial co-localization with acidified lysosomes was determined as the Overlap Coefficient (see *Text S1*).

### Infection of mice

Groups of naive mice (Female BALB/c mice 4–6 wk of age at 4/group) were infected with 1×10^6^ *M. tuberculosis* H37Rv via the tail vein. One group of mice was sacrificed 24 h later and lung homogenates were plated onto 7H11 agar plates for confirming infection. At ten days post infection, compound D4476 a final concentration of either 25 µM or 50 µM was injected into the tail vein of mice for treatment. The *i.v.* injection was repeated on seventh day of treatment. In addition the inhibitor was also given intraperitoneally on the third, fifth, tenth and twelfth day after initiation of treatment. At fourteen days following treatment, mice were sacrificed by carbon dioxide narcosis.

For experiments involving the aerosol route of infection, mice (8 per group) were infected with H37Rv by delivering between 100–150 bacteria per lung – as determined by the culture of lung homogenates at 24 h later - during 30 min of exposure. At the relevant times after infection, mice were sacrificed by carbon dioxide narcosis. The lungs were perfused and removed aseptically and weighed. The lungs were then homogenized and dilutions of these homogenates were plated on 7H11 agar plates for subsequent enumeration of the CFU.

## Supporting Information

Text S1 Supplementary Methods
(0.05 MB DOC)

Figure S1 Effect of D4476 on the intracellular growth of mycobacteria in primary mouse macrophages. Panel A shows the growth profiles of the indicated strains of Mtb in peritoneal mouse macrophages. The protocol employed here was identical to that described for Figure 3C in the main text. For the experiment in Panel B, macrophages independently infected with the three mycobacterial strains were treated with three rounds of addition of either the vehicle only (Control), or with D4476 at a final concentration of 50µM. Additions were performed at 16, 40 and 64h post-infection. The CFU values obtained are expressed as a % of that in the control group and values are mean (±SD) of three experiments. Panel C shows extent of co-localization of PHK67-stained mycobacteria with acidified lysosomes (stained with Lysotracker) at 72h as described for Figure 2. For each of the isolates (indicated on the right) the results for treatment of infected cells either with vehicle only (Mock), or with D4476 (50 µM, Inhibitor) is shown (indicated on the left side of the panel). Images shown for each of these groups are those obtained for PHK67-labeled Mtb (Mycobacterium), acidified lysosomes (Lysotracker), or a merge of the two (Merge).
(1.80 MB TIF)

Figure S2 Infection of J774.1 cells infected with H37Rv. J774.1 cells were infected with H37Rv labelled with PKH67 at an MOI of 10 as described in Methods). Panels A, B and C show confocal image of J774.1 cells after 24, 36, and 72 hours of infection respectively. Serial confocal sections (0.5 µm thick) within a z-stack spanning a total thickness of 10–12 µm were taken in green and transmission channels simultaneously, using the motor drive focusing system. More then 150 cells were analysed. The merged pictures of single confocal planes are shown.
(5.78 MB TIF)

Figure S3 Efficiency of siRNA transfection and the resultant silencing of expression of target proteins in J774.1 cells. J774.1 cells were transfected with AllStars Negative Control siRNA, Alexa Fluor 488 Labeled (RNAi Human/Mouse starter kit, Qiagen) at a 10nM concentration using hiperfect transfection reagent (according to manufacturer's protocol). Cells were fixed at 6h and 36h post-transfection. Panel A and B shows the J774.1 cells after 6 and 36 hours of transfection respectively. Fixed cells were imaged by confocal microscope as described for Figure S2. Panel C shows the Western blot data obtained after transfection of J774.1 cells with specific siRNA to monitor protein knock-down. siRNA transfection conditions were the same as that used in the screen and samples were collected at 24, 36, 48 and 72 hours post-transfection. At these times, cells were lysed and proteins resolved by SDS-PAGE followed by immuno-blotting against the target protein. In these gels, GAPDH and/or PLCγ2 were also probed to serve as the loading control. Panel D shows the extent of siRNA mediated knock-down for representative proteins from the list of validated hits in H37Rv infected J774.1 cells. Here infected cells were transfected with the relevant siRNA as described for our screening protocol (Methods) and protein levels measured by Western blot in cell lysates obatined at 48 hours. For TGFβRI, and CSNK1d results obtained with siRNA pools employed both in the primary (1) and validation (2) screen are shown. Panel E show the dose-dependent effects of siRNA treatment for TGFβRI and CSNK1d. Panel F shows the effects of variations in the extent of TGFβRI silencing, on the pathogen load. Infected cells were treated with the indicated doses of TGFβRI-specific siRNA, and the consequences on intracellular mycobacteria load was the determined. Results expressed in terms of % reduction in CFU obtained, from that in infected cells treated with GFP-specific siRNA. Panel G compares the extent of silencing between the pool of two siRNA employed in the primary screen (Pool), and the individual constituents of the pool (Oligo 1 and Oligo 2). Results for both CSNK1d and TGFβRI are shown.
(5.25 MB TIF)

Table S1 Screening Data for Kinase-Phosphatase siRNA Library. Primary screening data for H37Rv survival in J774.1 cells is presented in sheet 1 of Table S1. siRNA against each target gene was tested in duplicate, and data readings were obtained at two different dilutions for each. Here, we took care to ensure that both sets of the duplicate were not placed in adjacent wells of the plate. Instead, they were widely separated either across the wells of a given plate, or between two separate plates. Data for the two replicates are shown as CFU1 and CFU2 (×10^3^). The column control mean 1 and control mean 2 represents average control readings for each individual set, while SD1 and SD2 are their respective standard deviations. There are more than one control values for some plates because the whole plate was not set up at the same time in many instances. Deviation 1 and Deviation 2 are deviation of individual values from the control mean in terms of respective fold SD. Only those siRNAs giving an average deviation of value greater than 2 (positive or negative) in both replicates was taken to the next step for revalidation using siRNA sequences from a different source. While selecting average deviation greater than 2, we noticed that many of them showed large deviation between the two individual deviations. Nonetheless, they were also included in the primary hit list in order to avoid false negatives. Many spurious selections at this stage would later get filtered out in the revalidation due to more stringent selection criteria (more than 50% increase or decrease from the control). More than 50% criteria is stringent because most of the times the SD for a control value ranged between 10–20% of control mean, thereby selecting even those genes where the effect was less than 50%. Sheet 2 of the table contains the list of validated hits along with their values (percent reduction for both primary and validation, while average CFU for validation) and siRNA sequences used for validation experiment. For some genes for which siRNAs were not available in Sigma library (pool of three, columns F, G and H), we used siRNA pools from Dharmacon (pool of four, columns F, G, H and I).
(0.29 MB XLS)

Table S2 Cell Viability by MTT assay. Viability of H37Rv-infected and uninfected J774.1 cells was assessed at 90 hours after treatment with the siRNA pools for the 41 validated targets by the MTT assay (detail protocol in Methods). The average value for the MTT assay (mean of three) along with fold SD deviation from the control mean is shown for both infected and uninfected condition.
(0.02 MB PDF)

Table S3 Functional Class and disease association of ‘Hits’. ‘Hits’ identified in this study were searched extensively for their various GO class association, thereby summarizing the potential set of biological functions that they may be involved in regulating. We also examined for any known disease association for these ‘hits’ and this information is included in the table.
(0.04 MB XLS)

Table S4 Gene expression data for the validated targets. Hits filtered out after validation was checked for their expression level in J774.1 cells using one-color microarray-based gene expression analysis performed by hybridizing against a mouse whole genome array consisting of probes for 44,000 genes (Agilent). Values equal to or above 100 are considered as expressed. Expression levels were monitored at 16, 48 and 96 hours post infection.
(0.03 MB PDF)
