# Supplementary material for: Identification of Host-Dependent Survival Factors for Intracellular Mycobacterium tuberculosis through an siRNA Screen
Source: PLoS Pathog. 2010 Apr 15;6(4):e1000839. doi: 10.1371/journal.ppat.1000839 (PMC2855445; doi:10.1371/journal.ppat.1000839)
Supplement: Text S1 — Supplementary Methods (0.05 MB DOC) [file ppat.1000839.s001.doc]

**Text S1:**

**Materials and Methods.**

**Cells and culture.**

The murine macrophage cell line J774.1 (American Type Culture Collection) was used in this study. J774.1 cells were cultured in RPMI 1640 (Gibco Laboratories) supplemented with 10% FCS (Hyclone) and were maintained between 2 and 10 X 10^5^ cells per ml at 37^o^C in a humidified, 5% CO_2_ atmosphere. Before infection cells were plated in 96 well plates at 2.5 X 10^4^ cells per well overnight.

**siRNA library**.

Mouse Phosphatase siRNA set V 1.0 and Mouse kinase siRNA set V 1.0 library from Qiagen were used for the study. For validation experiments siRNAs against the kinases were obtained from Sigma siRNA (MISSION siRNA Mouse Kinase Panel), whereas the phosphatase-specific siRNAs were procured from Dharmacon (SMARTpool).

**Bacterial cultures.**

Virulent *M. tuberculosis* H37Rv and the two multi-drug resistant strains 1934 (a kind gift from Dr. Sarman Singh, AIIMS, New Delhi) and JAL 2261 (a kind gift from Dr. V. M. Katoch, Jalma) were used for the study. All experiments with M. tuberculosis were performed in a Biosafety Level III facility for the safe handling of pathogenic tubercle bacilli. Bacteria were grown in Middlebrooke 7H9 broth (Difco) supplemented with 10% ADC (Becton Dickinson), 0.4% Glycerol and 0.05% Tween 80 until the mid-log phase. The bacteria were then harvested, washed with RPMI with 10% FCS and resuspended in the same buffer. The suspension was dispersed by aspiration five times each with a 24- and then a 26-gauge needle, followed by an additional dispersion for 15 times through a 30-gauge needle. This was then vortexed until no bacterial clumps were detectable, and the dispersed bacteria were allowed to stand for 5 min. The upper half of the suspension was then used for the experiments. Bacteria were quantified by measuring the absorbance at a wavelength 600nm (0.6 O.D. corresponds to ~100 X 10^6^ bacteria).

**Infection of cells with Mtb.**

J774.1 cell were infected with tuberculosis bacteria at an MOI of 10 (i.e. 10 bacteria per cell). Cells were infected with bacteria in antibiotic free RPMI supplemented with 10% FCS. After adding bacteria, culture plates were centrifuged at 700 rpm for 5 min prior to incubation at 37^o^Cwith 5% CO_2_. After 4h, infected cells were washed twice with warm RPMI and replenished with complete RPMI containing 100μg/mL gentamicin for 2h to remove any remaining extra cellular bacteria and then in complete RPMI containing 10μg/mL gentamicin for rest of the experiment (see Fig. S2). In preliminary experiments we had first established that maintenance of infected J774 cells in the presence 10 μg/ml had no effect on intracellular mycobacterial growth, as determined by the subsequently obtained CFU values. Consequently, a gentamicin concentration of 10 μg/ml was taken for our experiments.

**siRNA transfection and CFU determination.**

After gentamicin treatment cells were transfected with siRNA at a final concentration of 100nM using the hiperfect transfection reagent (Qiagen) according to manufacturer’s protocol. At 48h later the cells were subjected to a second round of transfection with an identical concentration (100nM) of siRNA. The second round of siRNA treatment was done in order to prolong the duration of silencing of the target protein. In separate experiments during protocol standardization, we had first established that two rounds of either mock-transfection (i.e. hiperfect only), or with non-silencing siRNA (i.e. GFP-specific siRNA), had no significant effect on the subsequently obtained bacterial titers when compared with that obtained from parallel cultures where no transfection had been performed.

After an additional culture period of 42h, the cells were lysed in 50μl of 0.06% SDS for 10min at room temperature and dilutions were prepared. Undiluted cell lysate, and lysate dilutions to 1:10 and 1:100 were each plated separately in duplicate sets on 7H11 agar plates supplemented with OADC (Becton Dickinson) and 0.5% glycerol. Square plates were used for the plating, by the track dilution method in which 10μl of each dilution were spotted on one side of square plate. The plate was then tipped onto its side (at a 45^o^ -90^o^ angle), and the spots are allowed to gently flow in parallel tracks along the agar surface. The plates were then allowed to dry and subsequently incubated in a humidified incubator at 37^o^C. Colonies were first counted on 14^th^ day, and then again on the 21^st^ day of the incubation.

**MTT Assay.**

Cells were seeded in 96 well plates and siRNA transfections performed either in uninfected, or in infected cells as described above. At 90h later, the cells were washed with phenol red-free RPMI following which 100μL of fresh RPMI (serum-free) was added to each well. Subsequently, 20μl of MTT (Sigma) (5μg/μL) was added and the cells were incubated for 45min at 37^o^ C. Cells were then solubilized in DMSO (100μ), the formazan crystals dissolved by pipetting, and the absorbance measured at a wavelength of 560nm.

**Optimization of the experimental protocol.**

The above procedure was arrived at after a detailed standardization exercise where the individual steps involved were optimized as follows:

*Number of cells* *plated/well***:** This parameter was investigated with the objective of achieving conditions where the viability of the cell monolayer was optimal under conditions of both infection, as well as siRNA treatment and that cells should remain in a monolayer till the 5^th^ day. For this, experiments were performed in which cells were seeded at varying numbers ranging from 5000 up to 30,000 per well, and a seed density of 25,000 cells/well gave the best results.

*siRNA transfection conditions:* The conditions for siRNA transfection were optimized on cells that were plated as described above. In the first step we employed an Alexa 488-labeled siRNA to confirm that our transfection conditions yielded a transfection efficiency of >98% (Figure S3). To next define the silencing conditions, we chose siRNAs against six different target proteins (PKD, PKCδ, ERK-1/2, CaMKII, p38 and Pyk2). In these experiments the effect of siRNA treatment on the levels of the target protein was determined by Western blot analysis in cell lysates (lysis buffer: 20mM HEPES, 10mM NaCl, 1.5nM MgCl_2_, 0.2mM EDTA, 0.5% Triton X-100, 0.5mM DTT, 1 mM sodium orthovanadate, 1 mM NaF, and a cocktail of protease inhibitors) obtained at 36h after transfection (Figure S3C). The first set of experiments using a range of siRNA concentrations (from 1 to 100 nM) identified a 100 nM concentration as optimal, yielding a reduction of between 70-90 % in the levels of the different proteins. In a subsequent analysis of the kinetics of silencing, again by Western blot analysis, we observed a general trend where the reduction in protein levels was detectable by 24h, with maximal inhibition being obtained between 36-48h post-transfection. Following this, the protein levels began to increase by 72h such that basal levels were restored by the 96h time point. We therefore decided to include a second round of siRNA so as to ensure a more prolonged down-regulation of the target protein. For this we tested the effects of the addition of the second round either at 36h or 48h after the first one. In three separate experiments we consistently obtained better results with the 48h addition wherein the period of maximal down regulation of the protein extended from 36 to 96h. Consequently, this was the protocol that was adopted for our screening exercise.

In subsequent experiments, by taking a group of the validated target-specific siRNAs (CHEK1, TGFβRI, ABL1, DAPK3, WEE1, PRKACB, DGKZ, ADRBK1, and CSNK1d), we also confirmed that target-specific silencing could be efficiently achieved in cells infected with H37Rv (Fig. S3D).

*Multiplicity of Infection (MOI):* To determine MOI required for achieving maximum infection without affecting cell viability, J774.1 cells were infected at varying MOIs of 1, 2, 5, 10, 20, 25. At 24h, 48h, 72h, and 96h after infection, cell viability was monitored with trypan blue, and the proportion of infected cells was determined by confocal microscopy (see below). The best results were obtained at the MOI of 10 where between 80-90% of the cells were found to be infected, with a cell viability of >80% at the 96h time point. Whereas cell viability was retained at the lower MOIs, the percentage of infected cells was greatly reduced. In contrast, although a >90% of infected cells was obtained at MOIs of 20 and 25, there was – however – a significant reduction in the number of viable cells. Consequently, an MOI of 10 was adopted for our siRNA screen.

*Determination of CFU values:* For deciding the optimal dilutions to be used for the measurement of CFU counts, infected cells (in 96 well plates) were lysed in 50μl of 0.06% SDS. Then, 10μl was taken either directly from the lysate (neat), or from five different dilutions prepared from it (i.e.10^-1^, 10^-2^, 10^-3^, 10^-4^, 10^-5^), and plated as described earlier. In these experiments very few colonies were detected from lysate dilutions of, or greater than, 10^-3^. As a result, either undiluted lysate, or lysate dilutions of 10^-1^and 10^-2^ were selected for our experiments.

**Isolation of RNA and gene expression analyses.**

J774.1 cells were plated in wells of a six-well plate (2 x 10^6^ per well), and infected with H37Rv using the procedure described above. These cells were then collected either at 16h, 48h, and 96h post-infection. In parallel, an identical number of uninfected cells were used as control. RNA from each of these groups was isolated with trizol. One-color microarray-based gene expression analysis was performed by hybridizing against a mouse whole genome array consisting of probes for 44,000 genes (Agilent). For each time point, the hybridization was performed on duplicate sets of cells. For uninfected cells we used A/P calls as an indicator of the presence of a given transcript. To define alterations in the level of a given transcript following infection, a gene was considered to be upregulated if the signal log ratio between the infected and the uninfected sample was higher than one (> 2-fold increase), and the detection P-value of the infected sample was >0.95. Similarly, a gene was considered to be downregulated if the signal log ratio was less than minus one (> 2-fold decrease), and the detection P value of the reference sample was >0.95. Further, only those genes that presented a consistent change in both the biological repeats were taken as being differentially expressed.

**Confocal microscopy.**

*Infection and staining of cells:* J774.1 cells were seeded onto #1 thickness, 12 mm diameter glass cover-slips pre-coated with fibronectin in 24-well tissue culture plates at a density of 0.7x10^5^ cells per cover slip. These cells were then infected with PKH67-labeled mycobacteria at an MOI of 10 (Sigma; labeled according to the protocol recommended by the manufacturer), and incubated for 4h at 37^0^ C in 5% CO_2_. Extracellular bacteria were removed by washing and the culture medium replaced with RPMI supplemented with 100 μg/ml gentamicin to eliminate any residual extracellular bacteria. Cells were incubated with 100nM of the acidotropic dye, Lysotracker Red DND-99, during the last hour of the 72h chase. This dye freely permeates the cell membrane and, upon protonation in acidic compartments, remains trapped in the acidified organelles. This probe is particularly useful for examining phagosome acidification in vesicles with a very limited luminal content, such as in the case of those encapsulating Mtb. (V.Deretic et al, 1998). The cells were then fixed with 4% para-formaldehyde, washed (PBS, 3 times) and mounted on slides with Antifade (Biorad).

*Image capture*: Stained cells were observed with a Nikon TE 2000E laser scanning confocal microscope equipped with 60X/1.4NA PlanApochromat DIC objective lens. PKH67 and Lysotracker were excited at 488 nm and 543 nm with an argon ion- and a Helium-Neon laser respectively. The emissions were recorded through emission filters set at 515/30; 605/75. Serial confocal sections (0.5 μm thick) within a z-stack spanning a total thickness of 10-12 μm were taken in individual channels green and red using the motor drive focusing system. Images were acquired with a scanning mode format of 512 x 512 pixels. The transmission and detector gains were set to achieve best signal to noise ratios and the laser powers were tuned to limit bleaching fluorescence. The refractive index of the immersion oil used was 1.515 (Nikon). All settings were rigorously maintained for all the experiments.

*Image analysis:* All images were quantified using Image-Pro Plus version 6.0, a commercially available software package from Media Cybernetics. The merged confocal images were deconvolved and subjected to co-localization analysis to determine the “Overlap Coefficient” as previously described (Manders et al, 1993)

Here S1 is the signal intensity of pixels in the first channel and S2 that of the pixels in the second channel. At least 30-40 cells (>150 phagosomes combined from at least 4 random fields) from 3 separate sets of slides were examined for the co-localization studies. All images are in the Tiff RGB 24 format. To reduce the unwanted background noise generated by the photomultiplier signal amplification, the image stacks were treated with 2-dimensional filters (Gaussian filtering).

**Treatment of Mtb-infected J774.1 cells with D4476.**

Cells were infected with the respective strains and D4476 (purchase from Tocris Biosciences) was added at the indicated concentrations 16h later. A second and third round of drug addition was performed at 40h and 64h respectively, after an additional culture period of 26h, cells were lysed and the residual bacterial loads determined as CFU counts.

**Quantitation of TGFβ1 in cell culture medium:**

J774.1 cells were infected with H37Rv as described above and supernatants were collected at 0h from uninfected cells and at 16h, 48h and 90h post-infection from infected cells. TGFβ1 concentration was then determined using TGFβ1 Lincoplex kit according to manufacturer’s protocol.

**Histochemical staining of mice lungs:**

Sections of mice lung were stained using Hematoxylin/eosin in one set and with Ziehl-Neelsen stain in another set. Images were acquired at X10, X40 and X100 using Nikon 50i microscope using coloured camera.

## Isolation, culture and identification of clinical isolates of Mtb.

The sterile body fluids Cerebrospinal fluid (CSF) and Lymph node aspirate (LNA)] were directly inoculated in to the MGIT-960 culture bottles and part of the specimens was stored for the DNA isolation at -70^0^C. Where as, the contaminated specimens such as sputum, stool, urine, ascitic fluid received were first decontaminated with NALC-NaOH (modified Petroff’s method) as reported earlier. In brief, about 5-6 ml of contaminated specimen (sputum, urine or stool) was mixed with an equal volume of 0.5% NALC – 4% NaOH mixture in 50 ml ridge capped round bottom processing tube, the mixture was vortexed and incubated at 37^0^C for 10 min. After incubation, mixture was neutralized with phosphate buffer (pH 6.8) up to a total volume of 50 ml followed by centrifugation at 8000 x g for 10 min. The supernatant was decanted and the pellet was resuspended in 2 ml of the phosphate buffer. From the decontaminated sample, 0.5 ml of the suspension was inoculated into the BACTEC^TM^ MGIT tubes and 0.1ml in L-J slants under sterile conditions in the biosafety cabinet type 2 (Kartos, India) and from rest of the sample, smears were made for the Ziehl Neelsen (ZN) staining from both the direct and decontaminated specimens.

The inoculated MGIT-960 tubes were loaded in the BACTEC^TM^ MGIT-960 system, and the growth was continuously monitored upto 42 days in the fluorescence units that flash positive after reaching a cutoff growth described by the manufacturer. The LJ slants were incubated at 37^0^C for six weeks and checked every week for the growth. Positive signaled BACTEC^TM^ MGIT-960 tubes and colonies on Lowenstein-Jensen medium were confirmed for the presence of mycobacteria by microscopic examination of the smears prepared from these cultures. Positive cultures with no acid-fast bacilli, were also checked with Gram’s staining without concentration, and if showed other bacteria, these were considered contaminated and decontaminated as per the protocol mentioned above. If negative in both Gram and ZN staining, these were further incubated for a maximum period of 60 days or till the growth appeared in the medium. The *Mycobacterium* species isolated from the clinical specimens were identified phenotypically and by biochemical tests including heat stable catalase, nitrate, niacin and aryl-sulphatase test and finally confirmed by multiple molecular methods including the species specific PCR, Accuprobe^TM^ MTB complex detection test (Gen-Probe Inc, San Diego, CA 92121) and DNA sequencing, details of which are published elsewhere (Singh *et al.* Jpn. J. Infect. Dis. 60: 14-18 (2007); Gopinath *et al.* , Eur. J. Clin. Microbial. Infect. Dis. 27: 423-431 (2008); Gopinath and Singh, Int. J. Infect. Dis. In press, 2008).
