# Supplementary figures and images for: Identification of Host-Dependent Survival Factors for Intracellular Mycobacterium tuberculosis through an siRNA Screen
Source: PLoS Pathog. 2010 Apr 15;6(4):e1000839. doi: 10.1371/journal.ppat.1000839 (PMC2855445; doi:10.1371/journal.ppat.1000839)

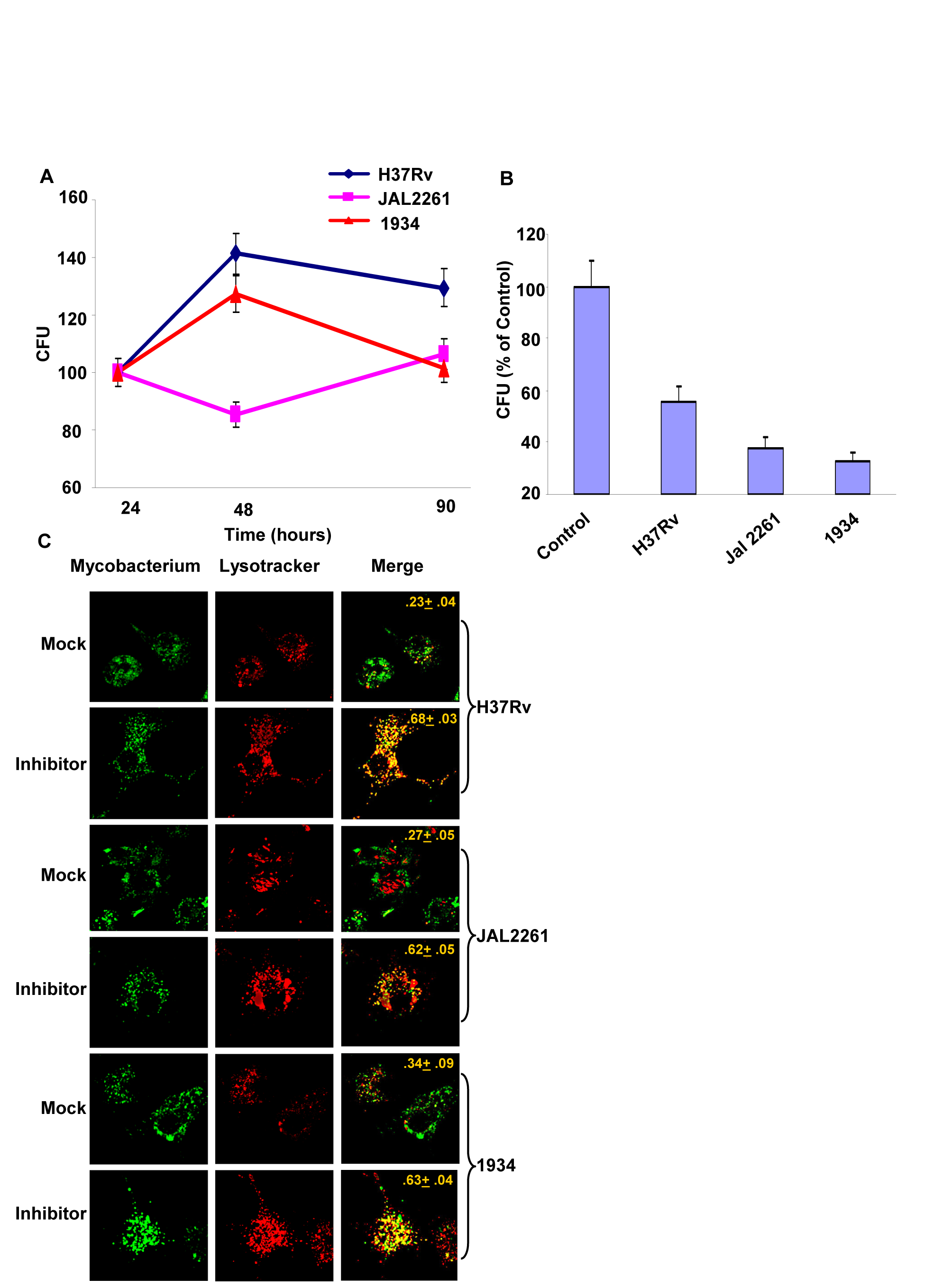

Supplement: Figure S1 — Effect of D4476 on the intracellular growth of mycobacteria in primary mouse macrophages. Panel A shows the growth profiles of the indicated strains of Mtb in peritoneal mouse macrophages. The protocol employed here was identical to that described for Figure 3C in the main text. For the experiment in Panel B, macrophages independently infected with the three mycobacterial strains were treated with three rounds of addition of either the vehicle only (Control), or with D4476 at a final concentration of 50µM. Additions were performed at 16, 40 and 64h post-infection. The CFU values obtained are expressed as a % of that in the control group and values are mean (±SD) of three experiments. Panel C shows extent of co-localization of PHK67-stained mycobacteria with acidified lysosomes (stained with Lysotracker) at 72h as described for Figure 2. For each of the isolates (indicated on the right) the results for treatment of infected cells either with vehicle only (Mock), or with D4476 (50 µM, Inhibitor) is shown (indicated on the left side of the panel). Images shown for each of these groups are those obtained for PHK67-labeled Mtb (Mycobacterium), acidified lysosomes (Lysotracker), or a merge of the two (Merge). (1.80 MB TIF) [file ppat.1000839.s002.tif]

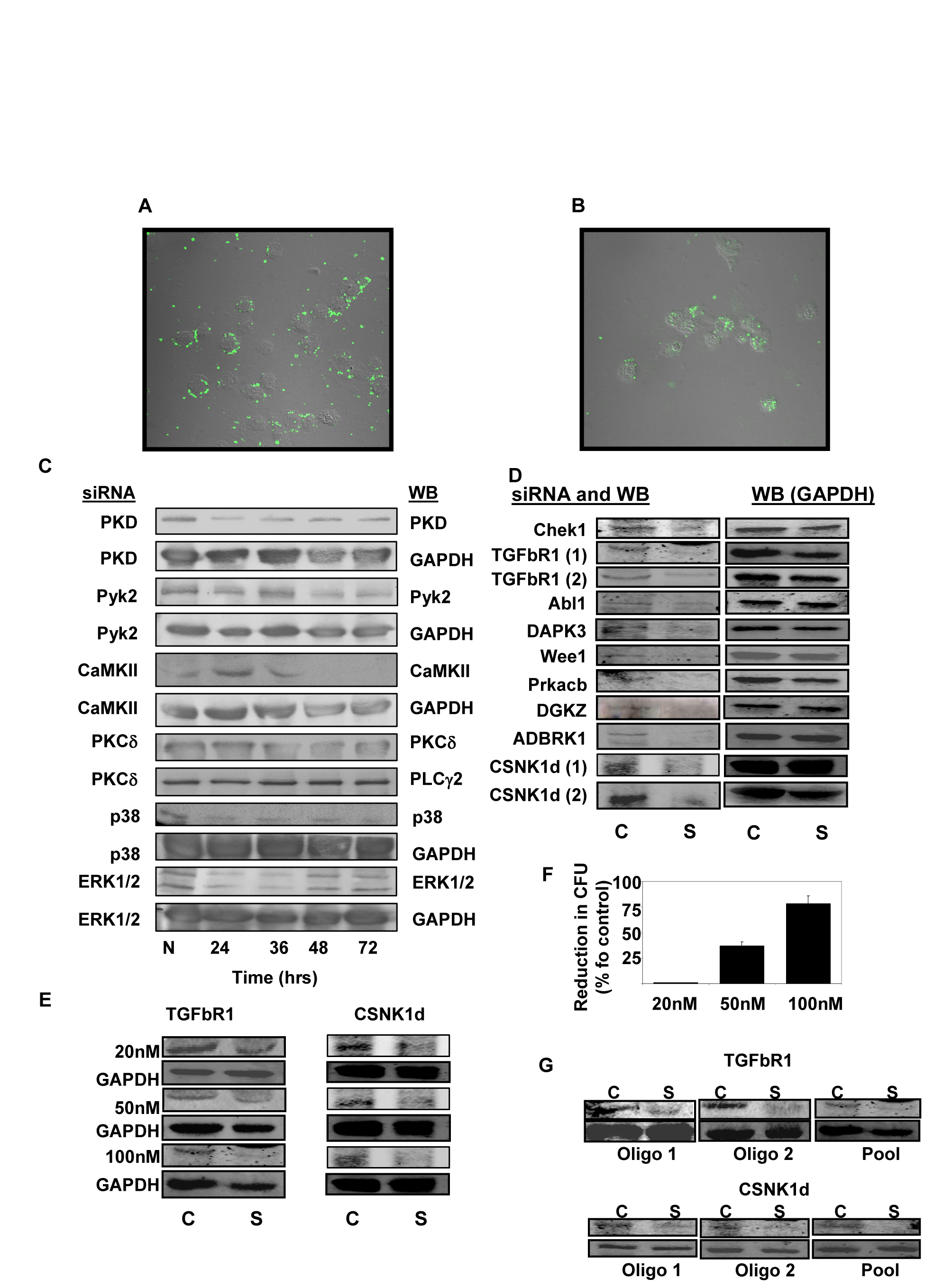

Supplement: Figure S3 — Efficiency of siRNA transfection and the resultant silencing of expression of target proteins in J774.1 cells. J774.1 cells were transfected with AllStars Negative Control siRNA, Alexa Fluor 488 Labeled (RNAi Human/Mouse starter kit, Qiagen) at a 10nM concentration using hiperfect transfection reagent (according to manufacturer's protocol). Cells were fixed at 6h and 36h post-transfection. Panel A and B shows the J774.1 cells after 6 and 36 hours of transfection respectively. Fixed cells were imaged by confocal microscope as described for Figure S2. Panel C shows the Western blot data obtained after transfection of J774.1 cells with specific siRNA to monitor protein knock-down. siRNA transfection conditions were the same as that used in the screen and samples were collected at 24, 36, 48 and 72 hours post-transfection. At these times, cells were lysed and proteins resolved by SDS-PAGE followed by immuno-blotting against the target protein. In these gels, GAPDH and/or PLCγ2 were also probed to serve as the loading control. Panel D shows the extent of siRNA mediated knock-down for representative proteins from the list of validated hits in H37Rv infected J774.1 cells. Here infected cells were transfected with the relevant siRNA as described for our screening protocol (Methods) and protein levels measured by Western blot in cell lysates obatined at 48 hours. For TGFβRI, and CSNK1d results obtained with siRNA pools employed both in the primary (1) and validation (2) screen are shown. Panel E show the dose-dependent effects of siRNA treatment for TGFβRI and CSNK1d. Panel F shows the effects of variations in the extent of TGFβRI silencing, on the pathogen load. Infected cells were treated with the indicated doses of TGFβRI-specific siRNA, and the consequences on intracellular mycobacteria load was the determined. Results expressed in terms of % reduction in CFU obtained, from that in infected cells treated with GFP-specific siRNA. Panel G compares the extent of silencing b [file ppat.1000839.s004.tif]
