# Supplementary material for: Identification of Host-Dependent Survival Factors for Intracellular Mycobacterium tuberculosis through an siRNA Screen
Source: PLoS Pathog. 2010 Apr 15;6(4):e1000839. doi: 10.1371/journal.ppat.1000839 (PMC2855445; doi:10.1371/journal.ppat.1000839)
Supplement: Table S2 — Cell Viability by MTT assay. Viability of H37Rv-infected and uninfected J774.1 cells was assessed at 90 hours after treatment with the siRNA pools for the 41 validated targets by the MTT assay (detail protocol in Methods). The average value for the MTT assay (mean of three) along with fold SD deviation from the control mean is shown for both infected and uninfected condition. (0.02 MB PDF) [file ppat.1000839.s006.pdf]

Supplementary Table 2

UnInfected			Infected		
Gene Symbol	Average	Fold Deviation*	Gene Symbol	Average	Fold Deviation*
Aatk	0.22345	-0.983167051	Aatk	0.11485	0.050952592
Abl1	0.2608	-0.363136838	Abl1	0.0988	-1.371289322
ADCK4	0.21055	-1.197314032	ADCK4	0.11175	-0.223748339
Adk	0.3283	0.757399691	Adk	0.09845	-1.402303943
Adrbk1	0.1963	-1.433871744	Adrbk1	0.0975	-1.486486486
Atp11a	0.24485	-0.62791547	Atp11a	0.0967	-1.557377049
Atp13a2	0.26695	-0.26104351	Atp13a2	0.12565	1.007975188
Atp1a3	0.2464	-0.602184631	Atp1a3	0.10765	-0.587062472
Atp2c1	0.2454	-0.618785172	Atp2c1	0.1055	-0.77758086
BTK	0.2395	-0.716728365	BTK	0.1184	0.365529464
CaMK2b	0.18805	-1.570826209	CaMK2b	0.09895	-1.357997342
Ccnd1	0.2103	-1.201464168	Ccnd1	0.11305	-0.108551174
Cdc25a	0.3217	0.647836119	Cdc25a	0.13365	1.716880815
Cdc25b	0.2276	-0.914274805	Cdc25b	0.11735	0.2724856
Cdc25c	0.2024	-1.332608443	Cdc25c	0.12245	0.724412938
CDK10	0.21285	-1.159132788	CDK10	0.1011	-1.167478954
Chek1	0.2524	-0.502581384	Chek1	0.1151	0.073105893
Csnk1d	0.23025	-0.870283371	Csnk1d	0.1312	1.499778467
Csnk1e	0.1797	-1.709440728	Csnk1e	0.10565	-0.764288879
DAPK3	0.2297	-0.879413669	DAPK3	0.09795	-1.446610545
Dgkz	0.2697	-0.215392022	Dgkz	0.12515	0.963668587
Dusp12	0.2419	-0.676887067	Dusp12	0.109	-0.467434648
Dusp14	0.2407	-0.696807716	Dusp14	0.12705	1.132033673
Dusp6	0.29795	0.253573266	Dusp6	0.1192	0.436420027
Entpd3	0.2305	-0.866133236	Entpd3	0.1001	-1.256092158
IRAK1	0.2459	-0.610484902	IRAK1	0.09815	-1.428887904
Itpkc	0.2425	-0.666926742	Itpkc	0.11165	-0.232609659
LRRK2	0.2821	-0.009545311	LRRK2	0.10005	-1.260522818
MAP3K4	0.23075	-0.861983101	MAP3K4	0.1098	-0.396544085
Pfkfb2	0.22655	-0.931705374	Pfkfb2	0.10795	-0.560478511
Ppapdc1	0.2516	-0.515861817	Ppapdc1	0.10855	-0.507310589
Prkacb	0.1959	-1.440511961	Prkacb	0.10465	-0.852902082
Prkag3	0.2119	-1.174903302	Prkag3	0.1273	1.154186974
SRPK1	0.2118	-1.176563356	SRPK1	0.11105	-0.285777581
TGFBR1	0.2792	-0.057686881	TGFBR1	0.10885	-0.480726628
TIE1	0.2722	-0.173890669	TIE1	0.12025	0.52946389
Trpm7	0.28125	-0.023655771	Trpm7	0.12645	1.078865751
TSSK5	0.28715	0.074287422	TSSK5	0.1164	0.188303057
Uckl1	0.306	0.387207623	Uckl1	0.1223	0.711120957
ULK1	0.23585	-0.777320341	ULK1	0.12815	1.229508197
Wee1	0.2339	-0.809691396	Wee1	0.1098	-0.396544085
Control	0.282675	0	Control	0.114275	0
ControlSD	0.0602		ControlSD	0.011285	
			Control (Uninfected)	0.13225	

* Fold Control SD from Control Mean
