# Supplementary material for: Identification of Host-Dependent Survival Factors for Intracellular Mycobacterium tuberculosis through an siRNA Screen
Source: PLoS Pathog. 2010 Apr 15;6(4):e1000839. doi: 10.1371/journal.ppat.1000839 (PMC2855445; doi:10.1371/journal.ppat.1000839)
Supplement: Table S4 — Gene expression data for the validated targets. Hits filtered out after validation was checked for their expression level in J774.1 cells using one-color microarray-based gene expression analysis performed by hybridizing against a mouse whole genome array consisting of probes for 44,000 genes (Agilent). Values equal to or above 100 are considered as expressed. Expression levels were monitored at 16, 48 and 96 hours post infection. (0.03 MB PDF) [file ppat.1000839.s008.pdf]

Supplementary Table 4

GeneAccession	GeneSymbol	GeneName	Basal Expression	16 hrs (Fold Change)	48 Hrs (Fold Change)	96 Hrs (Fold Change)
NM_029097	Atp13a2	ATPase type 13A2	**22525.5**	-0.42780805	-0.69441843	-0.47629356
NM_144921	Atp1a3	ATPase, Na+/K+ transporting, alpha 3 polypeptide	**232**	0.9289324	0.24090481	0.4033363
NM_008825	Pfkfb2	6-phosphofructo-2-kinase/fructose-2,6-biphosphatase 2	**221.5**	-0.56791496	-0.27188158	-0.8465917
NM_134079	Adk	adenosine kinase	**5666.5**	0.40093446	-0.5788436	-0.60458136
NM_015804	Atp11a	ATPase, class VI, type 11A	**1694**	-0.5102148	0.10855389	-0.050807238
NM_181593	Itpkc	inositol 1,4,5-trisphosphate 3-kinase C	**285.5**	0.054116488	0.19844246	0.6300795
NM_007828	DAPK3	death-associated kinase 3	**115.5**	0.48566866	-0.14225745	0.46091008
NM_175025	Atp2c1	ATPase, Ca++-sequestering	**1639.5**	0.39144945	-0.38347673	0.17407227
NM_009860	Cdc25c	cell division cycle 25 homolog C (S. cerevisiae)	**228.5**	-0.9709799	**-1.022527**	-0.6061163
NM_023117	Cdc25b	cell division cycle 25 homolog B (S. cerevisiae)	**4786**	0.16394782	-0.61674833	0.023393154
NM_026268	Dusp6	dual specificity phosphatase 6	**8721**	0.75669837	**1.2842727**	0.71861744
NM_183099	TSSK5	testis-specific serine kinase 5	**107**	0.6879909	0.2877829	0.18236232
NM_023173	Dusp12	dual specificity phosphatase 12	**8299**	0.6320169	-0.05355358	-0.14486742
NM_130863	Adrbk1	adrenergic receptor kinase, beta 1	**6475**	-0.12927794	-0.2696967	0.3518486
NM_008600	Mip	major intrinsic protein of eye lens fiber	**87**	0.5498791	-0.75016975	-0.60920477
NM_011100	Prkacb	protein kinase, cAMP dependent, catalytic, beta	**291.5**	-0.8386755	0.46411014	0.5839813
NM_133770	ADCK4	aarF domain containing kinase 4	**128**	0.049729586	-0.17793584	0.7098577
NM_027874	Csnk1d	casein kinase 1, delta	**8609**	**-1.2667077**	-0.049268246	0.21272898
NM_013482	BTK	Bruton agammaglobulinemia tyrosine kinase	**2341.5**	-0.21290088	-0.14018631	-0.5809326
NM_007377	Aatk	apoptosis-associated tyrosine kinase	**1483.5**	-0.10047412	**-1.7438765**	0.44411182
NM_009370	TGFBR1	transforming growth factor, beta receptor I	**585**	**1.0368283**	0.61356115	0.33505607
NM_025730	LRRK2	leucine-rich repeat kinase 2	**685**	0.89170885	-0.023011208	0.7484379
NM_001025438	CaMK2d	calcium/calmodulin-dependent protein kinase II, delta	**1702**	0.15199757	0.326607	0.13621569
NM_016795	SRPK1	serine/arginine-rich protein specific kinase 1	**951.5**	**-1.1252818**	0.33163047	0.14152813
NM_007658	Cdc25a	cell division cycle 25 homolog A (S. cerevisiae)	**209**	-0.51082206	0.83422256	-0.24863791
NM_178676	Entpd3	ectonucleoside triphosphate diphosphohydrolase 3	**61.5**	0.98613	0.3535986	0.41013575
NM_194444	CDK10	cyclin-dependent kinase (CDC2-like) 10	**3955**	-0.5590317	-0.47488737	0.11444616
NM_013767	Csnk1e	casein kinase 1, epsilon	**11157.5**	0.19787812	-0.21673155	0.5557418
NM_019819	Dusp14	dual specificity phosphatase 14	**74**	**1.5075603**	**1.2124228**	0.90275884
NM_008363	IRAK1	interleukin-1 receptor-associated kinase 1	**4023**	-0.5719049	-0.0874033	0.36228466
NM_153744	Prkag3	protein kinase, AMP-activated, gamma 3 non-catatlytic subunit	**83.5**	0.82417417	-0.03558898	0.6576135
NM_021450	Trpm7	transient receptor potential cation channel, subfamily M, member 7	**1840**	0.14491057	-0.39794827	-0.6653943
NM_009469	ULK1	Unc-51 like kinase 1 (C. elegans)	**1442.5**	0.4933226	0.35821962	0.83562374
NM_007631	Ccnd1	cyclin D1	**5899**	-0.02995181	0.03320217	-0.3657322
NM_011948	MAP3K4	mitogen activated protein kinase kinase kinase 4	**8596**	0.018889189	-0.44839668	-0.026413918
NM_009594	Abl1	v-abl Abelson murine leukemia oncogene 1	**250**	0.36668348	0.036658525	0.922277
XM_134059	Ppapdc1	phosphatidic acid phosphatase type 2 domain containing 1	**952.5**	0.41080308	0.22430897	0.12615585
NM_026765	Uckl1	uridine-cytidine kinase 1-like 1	**1036.5**	0.6423328	0.16378784	0.50451183
NM_011587	TIE1	tyrosine kinase receptor 1	**69.5**	**1.0498526**	0.28501058	0.35749078
NM_138306	Dgkz	diacylglycerol kinase zeta	**1622**	-0.4592116	0.27191257	0.8146763
NM_009516	Wee1	wee 1 homolog (S. pombe)	**115.5**	-0.61166525	-0.10788894	**-1.5683637**
NM_007691	Chek1	checkpoint kinase 1 homolog (S. pombe)	**789**	-0.121880054	-0.44817352	-0.9214654
